# How Pre-tenure and Tenured Faculty Can Engage Undergraduates in Publishable Research

**DOI:** 10.3389/fpsyg.2019.00111

**Published:** 2019-02-06

**Authors:** Albee Therese O. Mendoza, Jeannie A. Golden

**Affiliations:** ^1^Department of Psychology, Wesley College, Dover, DE, United States; ^2^Department of Psychology, East Carolina University, Greenville, NC, United States

**Keywords:** career stage, pre-tenure faculty, tenured faculty, early career faculty, late career faculty, publishing, undergraduates

Differences in career stage may influence work stress and job satisfaction (Olsen and Crawford, 1998), which in turn can impact attitudes about recruiting, managing, and mentoring undergraduates in publishable research endeavors. Written from the perspectives of a pre-tenure faculty member (i.e., in 4th year) at a primarily teaching institution (PTI) and a tenured faculty member (i.e., in 38th year) from a large research university (RU), this paper discusses obstacles faced by professors at different career stages and institutions while working on publishable research with undergraduates as well as strategies to overcome these obstacles.

## Pre-Tenure Faculty

Early career psychologists (ECPs) are faculty members working in their academic position within 7 years and have not obtained tenure (Keeley et al., 2013). For ECPs in RUs/PTIs, an important first step is to ask senior faculty how mentorship and publication fit with the department's expectations (Crawford, 2013). In PTIs, the pressure to “publish or perish” is not as salient as in RUs; however, ECPs are still eager to collaborate and publish with students for various reasons. One motivation is to include publications in their evaluation portfolio.

However, ECPs may not have much experience with publishing in general. One strategy to increase their knowledge of the publication process and the quality of writing required by journals, ECPs can read resources tailored to writing publishable research reports (e.g., Carver, 1984; Fallon, 2016). To boost their publication knowledge as well as enhance their portfolios, ECPs can volunteer in journals with open calls for reviewers, with some (e.g., Psi Chi, 2018a) not requiring any publication experience. This may have an added benefit later on in that being a reviewer for a journal may lead to increased confidence when mentoring a student who is publishing in that journal.

As an extension of their publishing inexperience and because they may not have a reputation at their institution, ECPS may have trouble finding students to work with on publishable research. In RUs, projects are often mentor-centered and students may take on roles as research assistants. Thus, a strategy for ECPs in RUs and PTIs is to recruit students directly from the courses they teach. ECPs can also reach out to colleagues and have them send students their way. In PTIs, projects are often student-generated and perhaps faculty mentors do not have course releases to do research nor have research labs. Thus, a strategy is to seek out advanced graduate-school bound undergraduates (Starke, 1985) such as those in capstone courses or honor thesis classes and encourage them to collect data and publish their work with the ECP as mentor.

However, ECPs (perhaps due to their eagerness and inexperience) may have uninformed expectations. ECPs may trust senior-level honors students to complete tasks without much involvement, due to the expectation that these students possess a positive attitude, emotional maturity, and strong work ethic.However, students may not match the expectations nor demonstrate the behaviors needed for the publication process, so there may be disappointment, anger, and regret when a project does not get published. One strategy to clarify expectations/behaviors is to develop written research learning contracts (Mabrouk, 2003) which clearly describe and may include the objectives of both parties, the tasks involved, deadlines when tasks are due, what happens if deadlines are not met (e.g., will the project continue after graduation?), what behaviors are expected on both ends (e.g., how soon should emails be answered?), and what happens if behaviors are not demonstrated (e.g., how will this impact letters of recommendation?). Another strategy to set the stage from the get-go is to write an open letter (McGuire, 2008) to any potential student outlining expectations/behaviors. If a student is taking a course in which a publication-ready manuscript is the end product, a clear rubric (Clabough and Clabough, 2016) can outline tasks related to publication (e.g., read submission guidelines) as well as expectations for conduct (e.g., asked questions in a timely manner).

Another issue related to student expectations/behaviors is that ECPs may not yet have adopted their own managerial style (Crawford, 2013). They may not have learned the necessary skills in graduate school nor had enough opportunity to practice these skills. One strategy for learning a managerial style is to solicit input from senior colleagues, from within and outside the institution; and the earlier, the better (Ponjuan et al., 2011). Senior faculty can provide ECPs different models of what works best for them and for the institution when publishing with students (e.g., benefits of being more hands-off vs. more involved) as well as give concrete examples of what they did to help a student successfully publish their work. They can also direct ECPs to resources to help manage and mentor student researchers (e.g., Narendorf et al., 2015; Shanahan et al., 2015).

Due to the pressure of evaluation and the desire to impress their students and colleagues, ECPs may take on too much, especially in the years leading up to tenure. Professionally, a strategy to boost both scholarship and *teaching* in their portfolio is to integrate their research in their teaching. One way is to incorporate the data collected from their publishable research as class exercises on data analysis, APA style, ethics, etc. Another way is to teach courses that may provide the perfect arena for generating research based on the course content or the structure of the course, which then could spurn student interest in research and eventually recruit mentees. A strategy to boost both scholarship and *service* in their portfolio is for ECPs to become student organization advisors. An ECP can encourage students who have high academic standings such as those in Psi Chi (Lechago et al., 2009) to work together on a publishable research project. Personally, ECPs may feel that they need to prove their worth to others and thus, they may take things personally and believe that what their students do or do not do (i.e., successfully publish or not) is a reflection on their ability or ineptitude. One strategy is to reach out to peers, either within and/or outside the institution, who are trustworthy and like-minded. With this support system, ECPs can be honest about personal and professional challenges, commiserate on like experiences, and brainstorm solutions to problems. If talking about sensitive issues with colleagues within the department/institution is uncomfortable, another strategy is to participate in a formal mentoring program (e.g., Finley, 2018) and discuss the aforementioned issues with more seasoned mentors outside the institution.

## Tenured Faculty

Late-career faculty (LCPs) are faculty members working in their academic position for 20 or more years and have achieved tenure (Baldwin and Zeig, 2013). In RUs and some PTIs, post-tenure review serves as a motivator for LCPs to continue to be engaged in teaching, scholarship, and service activities. In terms of scholarship, LCPs may have more time to work with undergraduates on publications by having course preparations done. They may also have existing data that need to be analyzed, and students may be more committed to working on a publication if they do not have to collect data from scratch nor go through the IRB process.

Due to their established network, LCPs may have increased connection to funding sources. Einarson and Clarkberg (2004) found that outside funding increased the likelihood of faculty including undergraduates in their research. However, funding sources (e.g., Society for the Teaching of Psychology, 2018) may disappear after the early career “clock” has run out or after one gets tenure. Thus, it benefits LCPs to know what funding sources are available to them regardless of career stage. For example, many conferences have undergraduate research awards and/or venues specifically designed for student presentations (e.g., American Psychological Association, 2018). Additionally, funding for student-led research as well as travel to professional conferences may be available from funding through student organizations (e.g., Psi Chi, 2018b). LCPs in RUs and some PTIs may have access to participant pool management systems (e.g., SONA) and/or online survey methods (e.g., SurveyMonkey) paid for by either internal funds or external grants, which can support data collection and save time.

Due to their experience, LCPs in RUs/PTIs may use their time more efficiently when engaging undergraduates in publishable research by choosing students wisely. LCPs at RUs and some PTIs who have lost funding for costly graduate assistants can fill this gap with highly trained and skilled undergraduates. If undergraduates are identified early in their college years, they will actually be available longer than master's level students. As these undergraduates assist, a natural vetting process takes place. It is easy to identify those undergraduates who are organized, meticulous, timely, and committed to doing the job well and getting it done; and ultimately perfect candidates for co-publishing. In addition, LCPs in RUs/PTIs are probably more adept at instructing, guiding, scaffolding, and knowing when to cut their losses.

Since LCPs in RUs/PTIs may have demands on their time with more administrative and leadership responsibilities, they may not have as much contact with undergraduates, particularly if they are not teaching undergraduate classes. Strategies to overcome this lack of contact include developing a website for their research lab (more likely in RUs) or listing their research interests and previously completed published projects on a faculty website. LCPs in RUs and some PTIs who developed research labs, that are layered with both undergraduate and graduate students at various levels of their college years, can provide valuable and sustainable mentoring to undergraduates who are interested in and capable of publishing. Thus, in RUs where LCPs may not be teaching undergraduates, often their graduate students do teach undergraduate students and may inform these students about the research that they themselves are involved with in the lab of their faculty mentor. In RUs/PTIs, another strategy is being a guest speaker for introductory courses and student organizations (e.g., Psychology Club) and emphasizing the importance of research productivity in the difficult challenge of gaining admission to graduate school as well as the advantage of research-related skills (e.g., collaboration, project management) in the workplace. Students can also be reminded that engaging in publishable research with faculty can lead to stronger letters of recommendation.

Some LCPs, despite systemic disincentives and heavy workloads, still decide to mentor undergraduates in the research process. Individuals with high levels of job satisfaction and a strong commitment to their place of employment are more willing to voluntarily engage in activities outside their specific job-related duties (e.g., mentoring undergraduate students) if they perceive it as having relevance to their work (Mamiseishvili and Rosser, 2010). For LCPs in RUs/PTIs, perhaps a strategy to have high job satisfaction is to think back to previous students and see how engaging in publishable research has impacted their lives. Keeping memorabilia, cards, pictures, or gifts on display in the office can be reminders of rewarding work with students. For some LCPs, certain students may become close friends after graduation and keeping in contact with them may be another reminder of the positive impact of their job. Another strategy is to foster friendships with colleagues who themselves have an optimistic attitude and high commitment to the institution. Overall, as they reflect on their tenure at their institution, LCPs who have been teaching and conducting research for many years may well find altruistic motivations for mentoring undergraduates as they begin to focus on the legacy and lasting effects of their careers.

## Conclusion

Working on publishable research provides hands-on skill development and close relationships between the undergraduate students and their faculty mentor, which helps those students bring their career aspirations to fruition (Seymour et al., 2004). Given these significant benefits to undergraduate students, it is important to highlight factors (e.g., career stage) that improve faculty's capability and willingness to publish with these students. Though the perspectives shared in this article may have some limitations (e.g., do not include mid-career faculty, do not include male viewpoints), when faculty mentors, no matter the career stage, collaborate with undergraduate students in publishable research, these students reap the benefits including improved cognitive skills and work ethic, increased preparation for graduate school, better career planning, and higher rates of retention (Hunter et al., 2006).

## Author Contributions

AM conceptualized the manuscript, composed the ECP section, connected all parts of the paper, conducted the final edits, and completed the submission process. JG worked on the LCP sections and provided general feedback.

### Conflict of Interest Statement

The authors declare that the research was conducted in the absence of any commercial or financial relationships that could be construed as a potential conflict of interest.
